# Lighting Conditions Influence the Dynamics of Protease Synthesis and Proteasomal Activity in the White Rot Fungus *Cerrena unicolor*

**DOI:** 10.3390/biom10091322

**Published:** 2020-09-15

**Authors:** Anna Pawlik, Magdalena Jaszek, Anita Swatek, Marta Ruminowicz-Stefaniuk, Beata Ciołek, Andrzej Mazur, Grzegorz Janusz

**Affiliations:** 1 Department of Biochemistry and Biotechnology, Institute of Biological Sciences, Maria Curie-Skłodowska University, Akademicka 19 St., 20-033 Lublin, Poland; magdalena.jaszek@poczta.umcs.lublin.pl (M.J.); anita.swatek@poczta.umcs.lublin.pl (A.S.); gjanusz@poczta.umcs.lublin.pl (G.J.); 2 Institute of Biological Sciences, Maria Curie-Skłodowska University, Akademicka 19 St., 20-033 Lublin, Poland; ruminowicz@poczta.umcs.lublin.pl (M.R.-S.); beata.rola@poczta.umcs.lublin.pl (B.C.); 3 Department of Genetics and Microbiology, Institute of Biological Sciences, Maria Curie-Skłodowska University, Akademicka 19 St., 20-033 Lublin, Poland; andrzej.mazur@poczta.umcs.lublin.pl

**Keywords:** proteases, light, transcriptomes, fungi, *Cerrena unicolor*

## Abstract

Recent transcriptomic and biochemical studies have revealed that light influences the global gene expression profile and metabolism of the white-rot fungus *Cerrena unicolor*. Here, we aimed to reveal the involvement of proteases and ubiquitin-mediated proteolysis by the 26S proteasome in the response of this fungus to white, red, blue and green lighting conditions and darkness. The changes in the expression profile of *C. unicolor* genes putatively engaged in proteolysis were found to be unique and specific to the applied wavelength of light. It was also demonstrated that the activity of proteases in the culture fluid and mycelium measured using natural and synthetic substrates was regulated by light and was substrate-dependent. A clear influence of light on protein turnover and the qualitative and quantitative changes in the hydrolytic degradation of proteins catalyzed by various types of proteases was shown. The analysis of activity associated with the 26S proteasome showed a key role of ATP-dependent proteolysis in the initial stages of adaptation of fungal cells to the stress factors. It was suggested that the light-sensing pathways in *C. unicolor* are cross-linked with stress signaling and secretion of proteases presumably serving as regulatory molecules.

## 1. Introduction

Fungi have an extraordinary capacity of adaptation to often drastic changes in the environment and response to the surrounding milieu via signaling pathways activated by specific stimuli. Many of these stimuli may be interpreted as potential stressors. These factors include light, which affects many aspects of fungal life, that is, primary metabolism comprising intracellular protein turnover, developmental decisions, stress response and physiological adaptations as well as the circadian clock regulating the expression of a large portion of genes encoded in the genome. Signal transduction is mediated by transcription factors that reprogram gene expression to cope with environmental changes [1,2,3]. The numerous examples of light responses in fungi indicate the complexity of light sensing and signal transduction comprising several photosensory systems, which respond to different light intensities and colors [4]. It should be emphasized that there are significant differences in the photoresponse to different light wavelengths in fungi. For example, a dominant metabolic effect of blue light was observed in *Neurospora crassa* contrary to that in *Aspergillus nidulans,* which responds mainly to red light conditions [5].

UV radiation represents a major source of stress for the cell and, in addition, induces the formation of reactive oxygen species (ROS) that is, superoxide, hydrogen peroxide or hydroxyl radicals. All these chemical particles can damage various macromolecules, including lipids, proteins and DNA. In fungi, visible light also serves as a common signal promoting resistance to UV-mediated damage, namely direct DNA damage and oxidative stress [1]. It has been shown that MAPK signaling pathways are critical for the adaptation of fungal cells to environmental conditions and are known to participate in multiple-stress response mechanisms [6].

Selective ubiquitin/proteasome-mediated proteolysis is known to play a crucial role in the response of cells to various stresses and, together with other enzymatic activities, can be considered as an indicator of the level of cellular stresses [7,8,9]. The appearance of stress factors in the growth environment of the fungus directly affects the level of synthesis and degradation of proteins involved in basic metabolism and thus changes in the rate of intracellular protein turnover [10].

*Cerrena unicolor,* a wood-degrading basidiomycete, is the causative agent of extensive white rot. It is described as a producer of industrially relevant bioactive compounds with great potential in biotechnological processes, pharmacy and medicine [11,12,13,14]. The RNA-seq based transcriptomic approach followed by detailed biochemical and microscopic analysis performed recently has demonstrated that light significantly influences *C. unicolor* metabolism and morphology [15,16,17,18]. A complex cross-interaction of nutritional and light signals on *C. unicolor* growth and micromorphology was also postulated [16]. Moreover, a possibility of light-modified activity of acid proteases in this fungus was also proposed [19]. However, no comprehensive studies related to proteases and ubiquitin-mediated proteolysis by the 26S proteasome in response to light are available. Given the abilities of the fungus to degrade wood material and production of biotechnologically important compounds, better understanding of protease secretion may be extremely valuable.

The present study implicates overall proteolytic activity and ubiquitin-mediated proteolysis by the 26S proteasome in the response of *C. unicolor* to different lighting conditions. The global changes in the expression profiles of *C. unicolor* genes putatively involved in proteolysis in response to light were also determined and new insights into light-dependent stress response in this biotechnologically important fungus were provided. It was suggested that the light-sensing pathways in *C. unicolor* are cross-linked with stress signaling and secretion of proteases presumably serving as regulatory molecules.

## 2. Materials and Methods

### 2.1. Fungal Strain and Cultivation Conditions

The *C. unicolor* FCL139 strain from the culture collection of the University of Regensburg, Regensburg, Germany, deposited in the Fungal Culture Collection (FCL) of the Department of Biochemistry and Biotechnology, Maria Curie-Sklodowska University, Lublin, Poland, was used in this work. The stock cultures were maintained on 4% (*w*/*v*) malt extract agar (BD, Difco, Franklin Lakes, NJ USA) plates. The Petri dishes were inoculated with mycelia, incubated at 28 °C for 10 days and then used for seed culture inoculation. As an inoculum, ca. 5 mm^2^ of the mycelium were punched out with a sterilized cutter. Then, the mycelia were transferred into a 100 mL liquid Lindeberg-Holm (LH) medium [20]. The seeds were cultivated in darkness at 28 °C. Next, the ten-day-old mycelia were homogenized in a disperser homogenizer (IKA, Staufen, Germany) and used as a standard inoculum. Solid-state lignocellulose *C. unicolor* cultures were grown at 28 °C on 1 g of sterile ash sawdust (wood particles < 4 mm) soaked with 9 mL of distilled water in Erlenmeyer flasks placed in incubators (KT 115, Binder, Germany) equipped with illumination LED cassettes for 14 days. The following lighting variants were applied—white (4000–4750 K), green (510–520 nm), blue (465–470 nm) and red (620–625 nm) light and darkness. Continuous lighting conditions (20 lux) were provided throughout the entire period of *C. unicolor* cultivation. Light intensity was experimentally determined using TM-209M LUX/FC Multi-LED light meter (Tenmars, Taipei, Taiwan), enabling the compensation of light intensity when different light wavelengths are applied.

### 2.2. Analysis of the Expression Profile of Genes Coding for Putative Proteolytic and Proteasomal Activities

In search of the expression profile of genes coding for putative proteases and proteasomes, we reanalyzed the data on the differential gene expression during *C. unicolor* cultivation in various lighting conditions (darkness, white, red, blue and green light) obtained in the RNA-seq experiment and described elsewhere [15]. Based on the abundance of transcripts and isoforms of the same gene between mycelia grown in darkness (control) and each of the lighting variants, differentially expressed genes (DEGs) were identified. Individual genes and transcripts were automatically annotated on the basis of predicted sequence similarities to known proteins of the SwissProt and TrEMBL databases as well as the presence of conserved domains (PFAM database). To reveal the expression profile of genes coding for proteolytic and proteosomal activities, which were differentially expressed in the tested lighting condition, we focused on DEGs classified as proteases and proteosomes. Each peptidase was annotated and grouped based on the mechanism of catalysis and a unique MEROPS identifier was assigned [21].

### 2.3. Preparation of Crude Sawdust Enzymatic Extracts

Extracellular extracts were obtained from solid-state lignocellulose *C. unicolor* cultures as described previously [18]. For this purpose, 10 mL of deionized water was added to each Erlenmayer flask and sawdust medium overgrown with the mycelium was crushed with a glass rod. Next, the suspension was stirred gently for 5 min using a magnetic stirrer and the enzymatic extract was separated from the sawdust and mycelium by filtration using Whatman No. 1 qualitative filter paper.

Intracellular extracts were prepared using 4 g of ash sawdust overgrown with the mycelium homogenized on ice (4 min processing cycle of 30 s pulses per min, 80% amplitude) in 15 mL of ice-chilled distilled water in an ultrasonic homogenizer (Sonics & Materials Inc, Newtown, CT, USA). Immediately after sonication, each sample was filtered through Miracloth and the homogenates were centrifuged (10,000× *g*, 15 min, 4 °C). Clear supernatant fractions were then aliquoted, frozen and kept at −20 °C.

The post-culture fluids (extracellular extracts) and supernatants of mycelium homogenates (intracellular extracts) were used for all determinations of proteolytic enzymes. All enzymatic analyses were carried out on probes of biological material collected on the 5th, 7th, 9th, 11th and 13th day of *C. unicolor* cultivation in above mentioned lighting conditions.

### 2.4. Spectrophotometric Analysis of Extra- and Intracellular Proteolytic Activities

The acid and alkaline protease activities were measured at two pH values according to the Anson method [22] using hemoglobin as a substrate. The reaction mixture consisting of 0.2 mL of the biological sample was incubated for 60 min with 0.5 mL of 1% hemoglobin in 100 mM citrate-phosphate buffer (pH 3.5) and 100 mM Tris-HCl buffer (pH 8.5). Next, the reaction was stopped by addition of 2 mL 5% TCA (trichloroacetic acid). Undigested proteins were then centrifuged and the resulting peptides and amino acid residues were measured spectrophotometrically at 280 nm. Appropriate control probes were performed for a 0 min incubation time. The specific proteolytic activity was expressed as U/mg of protein (one unit of protease activity was defined as the amount of the enzyme producing a 0.01 increase in absorbance per minute).

### 2.5. Spectrofluorometric Microplate Analysis of Extra- and Intracellular Proteolytic Activities

The detection of the intra-and extracellular proteolytic activities of acid and alkaline proteases was carried out with the fluorescence microplate method based on the digestion of three fluorogenic substrates—BODIPY-BSA, heavily labelled bovine serum albumin (Thermo Fisher Scientific, Waltham, MA, USA), BODIPY-casein (Thermo Fisher Scientific, Waltham, MA, USA) and BODIPY-elastin (Thermo Fisher Scientific, Waltham, MA, USA) in different pH ranges (3.5 and 8.5). The reaction mixture contained 50 μg of each fluorogenic substrate, 50 μL of the biological sample and 100 μL of appropriate buffer (100 mM citrate-phosphate buffer, pH 3.5 and 100 mM Tris-HCl buffer, pH 8.5). The fluorescence increase was measured over 30 min. (excitation λ = 590 nm and emission λ = 620 nm) using a Tecan Infinite 200 Pro microplate reader (Tecan, Crailsheim, Germany). The specific activity of proteases was expressed as the increase in the relative fluorescence units (RFU) per minute per mg of protein.

All microplate measurements were performed using an Infinite 200 Pro microplate reader (Tecan, Crailsheim, Germany) in triplicates in three biological replications.

### 2.6. Separation and Peptidase Activity of Proteasomes

The crude intracellular extracts were concentrated using Vivaspin 20 (300,000 MWCO, PES) ultrafiltration units (Sartorius AG, Gottingen, Germany) at 4 °C. Retentates containing high molecular weight fractions were assayed for proteasome activities.

Peptidase activities of the isolated 26S proteasomes were detected by monitoring the cleavage of the fluorogenic peptide substrate Suc-LLVY-AMC (Suc-Leu-Leu-Val-Tyr-7-amido-4-methylcoumarin) for chymotrypsin-like activity. The modified stopping procedure [23,24] was performed as described previously by Staszczak [25]. Briefly, a total volume of 100 μL of assay mixtures containing a 26S proteasome solution, 100 μM of a respective peptide substrate (in DMSO), 100 mM Tris-HCl buffer (pH 8.0), 2 mM ATP and 5 mM MgCl_2_ were incubated at 37 °C for 2 h with mixing. The reactions were stopped by addition of 100 μL of 10% SDS (*w*/*v*) and 2 mL of 100 mM Tris-HCl buffer, pH 9.0. The fluorescence of the formed 7-amido-4-methylcoumarin (AMC) was quantified in a spectrofluorometer (FluoroMax-2, Horiba Scientific/Spex Industries, Edison, NJ, USA), with an excitation wavelength of 360 nm and an emission wavelength of 440 nm. The excitation and emission slits were 1 and 0.2, respectively. The amount of released AMC was calculated using a standard curve of AMC in 5% DMSO in 100 mM Tris–HCl buffer, pH 8.0. In each light conditions, an appropriate control experiment was also performed to subtract the fluorescence of the culture medium [26]. Specific peptidase activity was expressed in nanomoles of AMC released per milligram of protein per 2 h.

### 2.7. Protein Determination

The protein concentration was determined according to the Bradford method [27] with crystalline bovine serum albumin (BSA) as a standard. All assays were carried out in triplicate.

### 2.8. Zymographic Analysis of Proteolytic Activities

In order to perform the zymographic analysis, samples containing 5 μg of protein per lane were loaded onto 10% separating gel containing 0.3% gelatine and 4% stacking gel according to Laemmli [28]. The electrophoretic separation was performed in non-denaturing conditions at 4 °C and 145 V. Next, the gels were incubated for 18 h at 37 °C in 100 mM citrate-phosphate (pH 3.5) buffer or 100 mM Tris–HCl (pH 8.5) buffer to detect acid and alkaline proteases, respectively. Then, the gels were stained with Coomassie Brilliant Blue R-250. Protease activity was visible as white bands of digested gelatine of the protease substrate. The imaging and analysis of the electropherograms were performed using the G: Box gel documentation system (Syngene, Frederick, MD, USA). Zymographic analysis were performed in triplicates in three independent biological replications.

### 2.9. Statistical Analysis

All measurements were performed in triplicates in three independent biological replications. All results are expressed as the mean ± SD (standard deviation) from nine measurements (n = 9).

## 3. Results

### 3.1. Effect of Light on the Expression of Proteases. Identification of Transcripts Coding for Putative Proteins Related to Proteolytic and Proteasomal Activities

**Figure 1:**
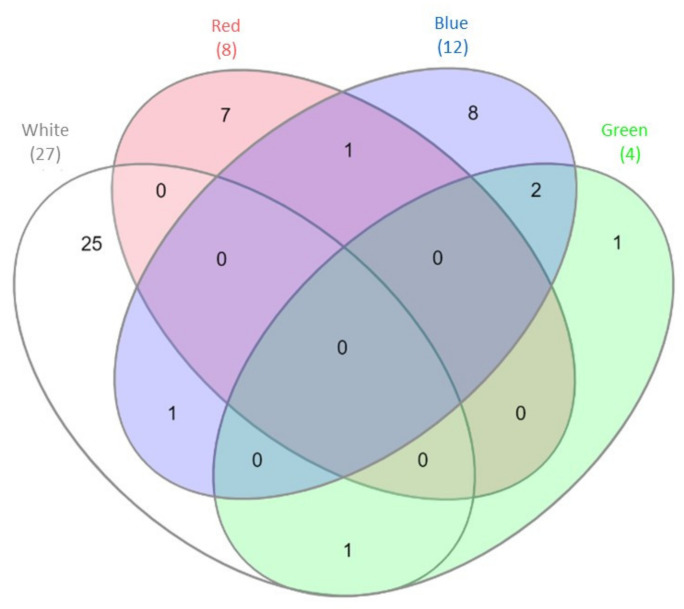
Venn diagram [29] demonstrating the number of differentially expressed genes (DEGs) engaged in proteolytic processes detected during *C. unicolor* growth in white light (light grey), green light (green), red light (red), blue light (blue) vs. dark conditions. The numbers of DEGs common/unique for the white, red, blue and green vs. dark conditions are indicated.

The results of the mRNA-Seq approach employed previously to gain insight into the gene expression profile of *C. unicolor* FCL139 during growth on ash sawdust in various lighting conditions comprising white, green, blue and red light and darkness as a control [15] allowed performing in silico analysis of the expression pattern of genes coding for putative proteases and proteasomal proteins. In general, the greatest number of differentially expressed genes (DEGs) engaged in proteolytic processes (10 up-regulated and 17 down-regulated) was observed when the fungus was grown in white light, compared to the control conditions. In contrast, the lowest number of DEGs (4 in total) was observed during *C. unicolor* growth in green light, compared to the control. It is worth noticing that from 1 to 25 transcripts coding for putative proteins with proteolytic and proteasomal activity were found to be specifically transcribed during cultivation of the fungus in one of the lighting conditions vs. darkness (Figure 1). The numbers of up- and down-regulated genes were comparable only in the white and green lighting variant. In the red light growth variant, the transcripts of up-regulated genes prevailed, whereas down-regulated genes predominated in the blue light variant, compared to the control (Table 1). Among the DEGs of *C. unicolor* cultivated in the tested lighting conditions, transcripts of genes coding for different families of proteolytic enzymes (aspartic, serine, cysteine and metalloproteases) were found (Table 1). White light repressed the expression mainly of genes encoding serine (XLOC_007328, XLOC_006577, XLOC_000740, XLOC_000739), cysteine (XLOC_007684, XLOC_001024) and metalloproteases (XLOC_001392, XLOC_003799). On the other hand, during fungus growth in the red light condition, up-regulation of the expression of metallo-(XLOC_000446, XLOC_006786, XLOC_006785) and all serine proteases (XLOC_006713, XLOC_010523) was observed. Surprisingly, all aspartic-type protease-coding genes (XLOC_007692, XLOC_010540, XLOC_007529) were down-regulated in all light variants, except for the red light, in which DEGs coding for acid proteases were not detected. The expression of genes coding for putative cysteine proteases was influenced only by white and blue light, whereas putative genes related to proteasomal activity were up-regulated in red light (XLOC_005808) and down-regulated in blue light (XLOC_000755, XLOC_007050, XLOC_007970, XLOC_011361).

### 3.2. Determination of Acid and Alkaline Proteolytic Activities in the Culture Medium and Mycelium of C. unicolor Cultivated in Different Lighting Conditions

In order to analyze the light-dependent changes in the extra- and intracellular proteolytic activities in the *C. unicolor* cultures, secretion profiles were examined using both natural and fluorogenic substrates at two pH values (3.5 for acid and 8.5 for alkaline proteases). Additionally, a zymographic analysis of the tested samples against gelatine as a substrate was performed. Extra-and intracellular extracts of *C. unicolor* cultured in darkness served as a control.

#### 3.2.1. Total Activity of Acid and Alkaline Proteases Using Hemoglobin as a Reaction Substrate

##### Acid Protease Activities

**Figure 2:**
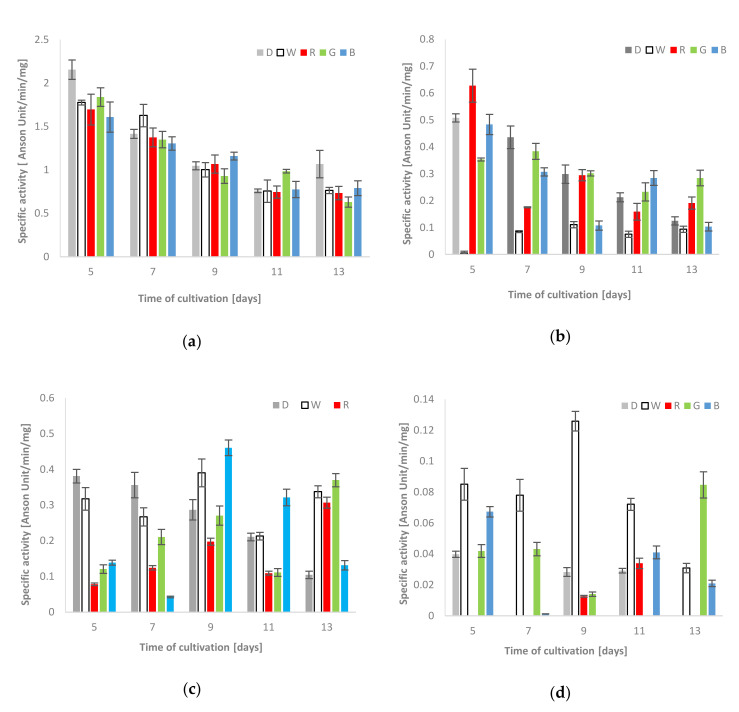
Total activities of acid [(**a**)–extra-and (**b**)–intracellular] and alkaline proteases [(**c**)–extra-and(**d**)–intracellular] using hemoglobin as a reaction substrate detected during *C. unicolor* growth in the dark (D), white (W), red (R), green (G) and blue light (B) conditions.

The spectrophotometric measurements showed that the proteolytic activity observed in the culture fluids was generally higher than that in the clear supernatants of mycelium homogenates. Moreover, the extracellular proteolytic activity in all lighting variants was lower (on the 5th and 13th day of fungus growth) or only slightly increased (white light on the 7th, blue - on the 9th and green - on the 11th day of the stress factor exposure) in comparison to the respective activities measured in the control cultures grown in darkness (Figure 2a). In the case of mycelium, the highest activity of acid proteases, that is, 0.63 units/min/mg, was observed on the 5th day of the fungus exposure to red light (Figure 2b). Moreover, a noticeable increase in acid protease activities, in relation to the control conditions, was also observed in the blue light conditions on the 11th day of the experiment (0.28 units/min/mg) and in the red and green lighting variant (0.19 and 0.28 units/min/mg, respectively) on the last day of the *C. unicolor* cultivation. A clear relationship between the decrease in the acid protease activity against hemoglobin and the time of fungal cultivation was observed in almost all experimental variants, except the intracellular white light variant, where the lowest intracellular activities of acid proteases, which did not exceed 0.11 units/min/mg, were noted throughout the entire period of *C. unicolor* growth (Figure 2b).

##### Alkaline Protease Activities

In general, the extra- and intracellular activities of alkaline proteases were variable and seemed to be dependent on the day of cultivation. The highest alkaline protease activity against hemoglobin in the fungal mycelium extract was observed in the white light condition (0.13 units/min/mg on the 9th day). The recorded activities were relatively higher than the control values throughout the entire period of *C. unicolor* cultivation. On the 7th day of the fungus growth, the proteolysis of the protein substrate was diminished and observed only in the white and green light conditions. The highest intracellular activities of alkaline proteases on the 13th day of *C. unicolor* incubation were detected in green light and reached 0.08 units/min/mg (Figure 2d). In turn, on the 5th and 7th day of fungus culturing, the control values measured for extracellular proteases were clearly higher. On the 9th day, the activities in cultures grown in white and blue light reached the highest values of 0.39 and 0.46 units/min/mg, respectively. Clear dominance of alkaline proteases in culture extracts from the blue light variant was observed on the 9th and 11th day. Furthermore, the activities obtained in the white, red and green light *C. unicolor* cultivation conditions on the 13th day clearly exceeded the control values and proved to be more than twice as high (Figure 2c).

#### 3.2.2. Total Activities of Acid and Alkaline Proteases Using BODIPY-BSA as a Reaction Substrate

##### Acid Protease Activities

The extra- and intracellular activities of acid proteases towards the BODIPY-BSA substrate were the highest (41.05 RFU/min/mg and 31.02 RFU/min/mg, respectively) on the 5th day of the *C. unicolor* cultivation in the red light conditions. Importantly, the extracellular activities in the blue and red light variants were comparable and both exceeded the control (the darkness) throughout the entire period of the fungus growth (except the 11th and 13th days in the green variant). The intracellular proteolytic activities obtained in the fungus cultivation in red light were also higher than the control values (Table 2). Almost similar trend was observed for the green light variant, except the first days of incubation, where the activities of acid proteases against the BODIPY-BSA substrate detected in darkness were higher. In addition, on the 9th and 11th day of fungus growth in the white light conditions vs. the control, a slight increase in the intracellular proteolytic activity was noted. A general downward trend in the activity of proteases with the time of fungal cultivation was observed (Table 2).

###### Alkaline Protease Activities

The highest activity of extracellular alkaline proteases in the extracts was found on the 5th day of *C. unicolor* growth in the red and blue light conditions and reached comparable values of 52.71 and 51.34 RFU/min/mg, respectively. In general, clear induction of extracellular protease activity (except the 5th day) was observed in relation to the control conditions (Table 2). Moreover, the strong stimulatory effect of green light conditions on the described biochemical parameter throughout the entire period of *C. unicolor* cultivation, which ranged from 39.11 to 17.69 RFU/min/mg, should be emphasized. As with the extracellular extracts, the intracellular filtrates showed the highest (37.99 RFU/min/mg) proteolytic activities against BODIPY-BSA in the red light condition on the 5th day of the experiment. In turn, the lowest intracellular activities of alkaline proteases were detected for the white-light fungus growth conditions vs. all the other lighting variants (Table 2).

#### 3.2.3. Extra–and Intracellular Elastase-Like Activities Detected Using BODIPY-Elastin as a Reaction Substrate in Alkaline Conditions

Obvious induction of the extracellular proteolytic activity was observed in the fungus cultivation in red and blue light and the highest elastase-like activities in these conditions were determined on the 5th day, that is, 72.61 and 68.93 RFU/min/mg, respectively. Although substantially lower proteolytic activities were noted in the other culturing variants, they were higher than in the control (Table 2). A very similar stimulatory effect was observed for the samples of mycelium extracts. Likewise, intracellular elastase-like proteolytic activities appeared to be light-dependent and strong stimulation by white, red, green and blue light was shown. However, this activity was definitely the highest for the fungus grown in red and blue light (58.74 and 57.69 RFU/min/mg, respectively) in comparison to the control and green light conditions (Table 2).

#### 3.2.4. Detection of Extracellular Caseinolytic Activities Using BODIPY-Casein as a Reaction Substrate in Alkaline Conditions

The extracellular caseinolytic activity of *C. unicolor* was diverse and variable but also appeared to be light-dependent. The highest activity was observed for the culture fluids of the fungus grown in the darkness, white and green light conditions and reached their maximum values on the 5th day of the experiment (156.62, 165.83 and 153.64 RFU/min/mg, respectively). The application of red and blue light during *C. unicolor* cultivation clearly inhibited the activity of caseinolytic proteases present in the examined extracts in comparison to the control (darkness). A decrease in the level of substrate digestion along with the culturing time was also noted (Table 2).

### 3.3. Zymographic Analysis of Proteolytic Activity in the Culture Fluid and Fungal Mycelium Using Gelatine as a Protein Substrate in Acidic Conditions

**Figure 3:**
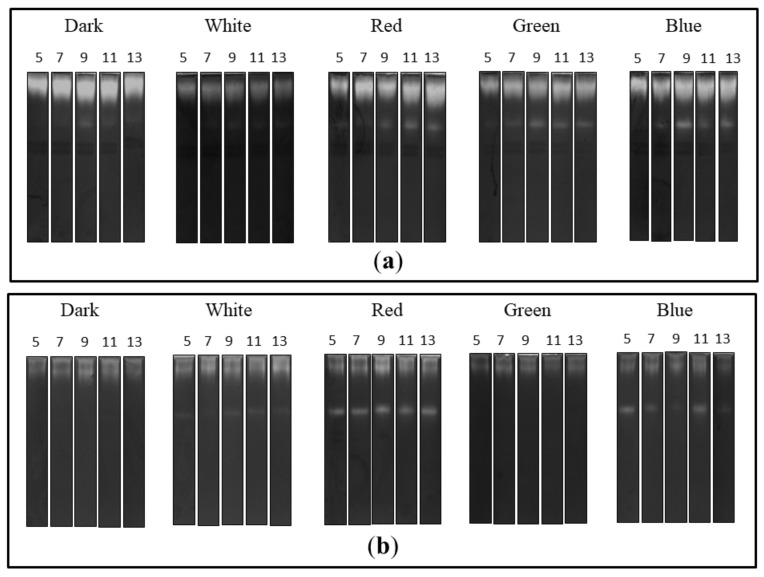
Zymograms of the activity of extra- (**a**) and intracellular (**b**) *C. unicolor* proteases detected in acid conditions during fungus growth in the dark (control), white, red, green and blue lighting conditions; for each light variant, the number of days of *C. unicolor* cultivation, after which the zymographic analyses were performed, is indicated.

The zymographic analysis of the extra- and intracellular fungal extracts showed the presence of bands indicating proteolytic activity in all tested preparations in acidic conditions (Figure 3). A clear increase in the gelatine digestion rate was observed in the case of the extracellular extracts obtained from fungal cultures growing in the red, green and blue light conditions. There was a characteristic appearance of additional rapidly migrating bands corresponding to protease activity, whose intensity increased markedly during the experimental period. Moreover, the intensity of bands indicating extracellular proteolytic activity of *C. unicolor* grown in white light was the lowest in relation to the other lighting variants, including the control (Figure 3a). Additional bands of proteolytic activity were also detected by native PAGE in samples of intracellular extracts prepared from the fungus cultured in the red and blue light conditions. The intensity of bands showing proteolytic digestion of the substrate was lower in the white light variant than in the case of the red and blue *C. unicolor* cultivation conditions; however, an additional activity band was detected in all these cases. Surprisingly, no fast migrating proteolytic bands were detected in the intracellular extract of the fungus cultivated in the green light conditions. The most intense activity bands throughout the entire experimental period were detected in the red light variant. In contrast, the lowest proteolytic activities in the tested intracellular samples were recorded in the darkness and green light conditions (Figure 3b).

### 3.4. Chymotrypsin-Like Activity of C. unicolor 26S Proteasomes

**Figure 4:**
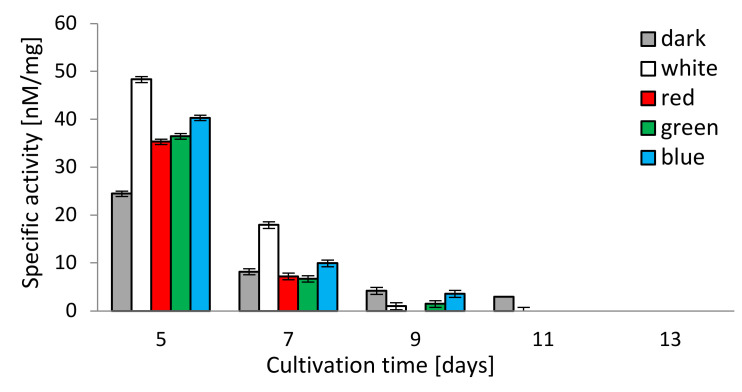
Chymotrypsin-like activity (CHT-L) of *C. unicolor* 26S proteasomes detected in the white, dark, red, blue and green light conditions.

The chymotrypsin-like activity (CHT-L) of the 26S proteasomes isolated from the mycelia of *C. unicolor* measured against the fluorogenic Suc-LLVY-AMC substrate was light-dependent as well. All lighting conditions stimulated the chymotrypsin-like activity of *C. unicolor* proteasomes, especially on the 5th and 7th day of the cultivation period, where the highest proteasomal activity (48.3 and 17.9 nM/mg, respectively) was detected in the white light conditions. In contrast, the lowest stimulation of the CHT-L activity of *C. unicolor* proteasomes was recorded in darkness throughout the entire period of cultivation. The highest CHT-L activities in the red, blue and green light conditions were measured on the 5th day of fungus growth and they reached 35.3, 40.3 and 36.4 nM/mg of protein, respectively. A decrease in the *C. unicolor* proteasomal activity over the cultivation time was observed and no CHT-L activity was detected in all lighting variants on the 13th day (Figure 4).

## 4. Discussion

In the environment, fungi face many potential adverse factors, such as temperature changes, UV radiation, starvation or osmotic stress. To survive and grow in such stressful conditions, these organisms have evolved multiple defense systems, some of which rely on enzymatic actions and are linked to strong regulation of metabolic pathways, including proteolysis [30]. Even a slight change in the lighting conditions of fungal cultures seems to induce a cellular response similar to that to other stress factors. Since stress adaptation is crucial for the survival process, the presence of a diverse photosensory machinery is advantageous in evolution [4].

The stress-inducing effect of various wavelengths of light on *C. unicolor* was demonstrated in our previous studies on the redox imbalance in fungal cells manifested in elevated catalase and superoxide dismutase activities [18]. One of the effects of the action of stressors inside cells is the simultaneous appearance of a large amount of functionally and structurally altered proteins and concomitant appearance of a whole group of chaperones such as various heat shock proteins [31], whose transcripts were also present in the transcriptome of *C. unicolor* mycelium growing in different lighting [15]. In stressful conditions, significant changes occur also in the rate of degradation and synthesis of individual proteins, which has a direct impact on the overall protein turnover in cells [10]. Therefore, the cell proteolytic system, which has major importance in restoring homeostasis by repairing or degrading damaged molecules, is remodeled [9,32]. Regulation of protein degradation and repair systems through numerous stress proteins, including heat shock ones, was postulated [31,33].

In this study, we demonstrated that the activities of extracellular and intracellular proteases of *C. unicolor* were regulated by light. Comprehensive phenotypic and transcriptomic analyses have already revealed that light exerted a global influence on the metabolism of this fungus [15,17]. However, the physiological significance of regulation of protease activities by light remains largely unclear and no attempts of studying light-dependent protease synthesis in fungi have been made to date. Our results indicate a clear influence of light on protein turnover and the observed qualitative and quantitative changes in the hydrolytic degradation of proteins catalyzed by various types of proteases. Here, genes coding for putative proteins with proteolytic and proteasomal activity were found to be specifically and differentially transcribed during fungus cultivation in one of the lighting conditions vs. darkness, suggesting that C. *unicolor* can respond to light wavelengths in various manners. Accordingly, the up-regulation of genes involved in protein turnover was demonstrated upon illumination of *T. reesei* mycelium [34].

The analysis of proteolytic activity associated with the 26S proteasome showed a marked increase in the rate of substrate digestion in the early stages of *C. unicolor* growth. These results indicate the key role of ATP-dependent proteolysis in the initial stages of adaptation of fungal cells to stress factors—in this case the changing light wavelength. The analysis of the *C. unicolor* transcriptome also suggests differences in the abundance of transcripts associated with the synthesis of ubiquitin, that is, a small molecule directly cooperating with the proteasomal complex towards degradation of intracellular proteins. Selective ubiquitin/proteasome-mediated proteolysis is known to play a crucial role in the response of white rot fungi to various stresses such as nutrient limitation, heat shock, ferulic acid and heavy metal [8,25,35]. Available data indicate involvement of the ubiquitin-proteasome pathway in the regulation of ligninolytic enzyme activities in starvation conditions and stress caused by the presence of Cd^2+^ ions [35].

In *C. unicolor*, light-dependent expression of intra- and extracellular proteases was detected, suggesting that changing light conditions can directly affect the level of proteolysis in fungal cells. A direct influence of light on acid protease activity has been reported previously for *C. unicolor*, *Phlebia lindtneri* and *Pycnoporus sanquineus* [36]. In *Aspergillus oryzae* cells, proteolytic activity showed sensitivity to light and production of respective enzymes was increased in darkness [37]. It was also found that darkness and constant lighting conditions significantly modified the activity of acid, neutral and alkaline proteases produced by *N. crassa* [38].

The analysis of the *C. unicolor* DEGs showed that genes encoding for putative aspartyl proteases were down-regulated in mycelium grown in white, blue and green light in comparison to darkness. However, the spectrophotometric and spectrofluorimetric measurements performed against synthetic and natural protein substrates using an aspartyl protease inhibitor—pepstatin revealed that it reduced the total proteolytic activity up to 85% (unpublished data), further indicating that aspartyl proteases dominate in the secretomes of the studied fungus. The analysis conducted in the present work showed very interesting correlations between the application of light and the level of proteolysis in acidic conditions. Surprisingly, the level of extra- and intracellular protease activity in acidic conditions was dependent on three variables—the phase of the fungal culture growth, the lighting variant used and the type of the protein substrate. Regardless of the substrate, that is, hemoglobin, gelatine or BODIPY-BSA, the cultivation of *C. unicolor* in red light significantly increased the activity of acid proteases in comparison to darkness, especially in the early stages of fungal growth. This trend was particularly discernible in the digestion of the natural substrates by intra- and extracellular proteases in the case of BODIPY-BSA. These results suggest a special relationship between the role of red light photoreceptors in the studied fungal cells and stimulation of acid protease activities. A similar relationship as in the case of *C. unicolor* proteases has already been observed for micromorphological features [16]. Moreover, the appearance of an additional band of acid protease activity in the zymograms of the intracellular preparations from the red and blue light culture variants and the extracellular samples of cells propagated in blue and red light seems to be interesting. This fact may be related to the stronger activation of proenzyme molecules (precursor forms of protease migrating more slowly in the electric field) into catalytically active hydrolases, the activity of which was revealed as an additional proteolytic band.

Serine (alkaline) proteases are generally considered as housekeeping enzymes involved in protein turnover, protein maturation, signal transduction and signal peptide cleavage. It was found recently that the general fungal lifestyle is reflected in the overall encoded serine protease repertoire [39]. However, it seems that genes coding for serine proteases in *C. unicolor* are related not only to the fungal ecology but also to growth in various lighting conditions. Serine protease-coding genes were found to play a protective role in oxidative stress response in *Clonostahys rosea* [40]. The activity of *C. unicolor* proteases in alkaline conditions was light- and substrate-dependent and appeared to be more stable than that of the acid proteases, which may be due to their regulatory role in fungal cells. Caseinolytic proteases, which are conserved across the tree of life and are involved in intracellular protein degradation (turnover) and homeostasis [39], were clearly inhibited in the examined extracts by red and blue light. In turn, the synthesis of elastase-like proteases, which act as a putative virulence factor in fungi [41], was increased in the same lighting conditions. For saprotrophic and parasitic organisms the ability to secrete proteases may be crucial for surviving and successful colonization of new ecological niches.

In fungi, the processes of protease synthesis and light sensing are linked to many metabolic pathways. Recent studies on regulation of protein phosphatases in *T. reesei* revealed dependence of protease production by Pty4, Pzl1 and Dsp1 phosphatases in a light dependent manner. Phosphatase-deficient mutant strains showed a more pronounced decrease in protease activity under constant light compared to darkness [42]. Post-transcriptional cross-regulation of protease synthesis in *T. reesei* was also observed in the case of cellulase production through the *clf1* gene (cellulase and light associated factor 1) involved in regulation of secreted protease activity [43]. It was also found that illumination significantly stimulated proteolytic activity in other *Trichoderma* strains and was dependent on the BLR1 and BLR2 receptors [44]. Such a complex picture of protease synthesis allows speculation about existence of similar cross-regulatory pathways in *C. unicolor*.

**Figure 5:**
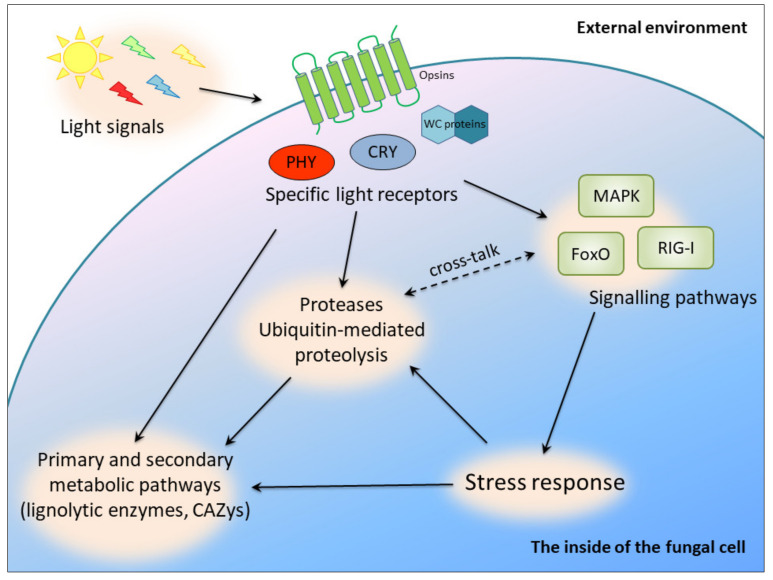
Putative light-dependent cross-regulatory pathways in *C. unicolor* involving proteases; PHY, phytochromes; CRY, cryptochromes; WC proteins, white collar proteins.

It has been proved that the light-sensing pathways and molecules are interlinked with stress signaling in many fungal species. A link between light and stress signal transduction was discovered in *Botrytis cinerea*, *Cryptococcus neoformans* and *Aspergillus nidulans* [4]. However, our current knowledge does not allow us to present a unified picture of light signaling in fungi. In *C. unicolor*, the blue light signal seemed to be transduced via FoxO (the “Forkhead box” (FOX)) to the processes of oxidative stress response and DNA repair, whereas white light may have exerted an effect on ubiquitin-mediated proteolysis. The latter process may also have been influenced by white light via an alternative signaling pathway involving the RIG-I-like receptor, whose expression was induced in the studied conditions [15]. It was also demonstrated that, at the transcriptomic level, white and blue light influenced mitogen-activated protein kinase (MAPK) pathways in *C. unicolor* [15], which constitute canonical signaling involved in the regulation of cellular differentiation and proliferation in eukaryotes. In turn, in *Candida albicans*, aspartic protease-mediated proteolytic cleavage of Msb2 mucin is required for activation of the MAPK pathway in response to environmental cues [45]. Stress-activated protein kinases (SAPKs), which are conserved MAPK signaling modules, integrate stress and light signals in *T. atroviride* [6]. Similar activation of MAPKs was also confirmed recently in *Aspergillus fumigatus* and *C. neoformans* [46]. This indicates complex regulation of metabolic pathways in fungi interlinked with light signaling and secretion of proteases serving as regulatory molecules (Figure 5).

## 5. Conclusions

In conclusion, microbial proteases are greatly influenced by physicochemical factors, including light and seem to fulfil very important and diverse regulatory roles. These enzymes are directly involved in the activation and integration of many metabolic pathways and are interlinked to the light-sensing pathways and molecules. The conducted research demonstrates a unique relationship between changes in lighting conditions and the level of intra- and extra-cellular proteolysis, including the activity of proteolytic enzymes from different catalytic groups into one scheme. This indicates a complex picture of molecular regulation exercised by proteases commonly produced in many fungal species. Bearing in mind the great biotechnological importance of proteases themselves and the fact that fungal proteases are a major bottleneck in industry for efficient production of proteins of interest, better understanding of its synthesis and secretion may be extremely valuable. Given the abilities of *C. unicolor* to degrade wood material and produce important enzyme-based compounds, the knowledge on the molecular regulation of proteases may be applied to improve the efficiency of biotechnological processes involving this fungus. However, further attempts should be made to investigate the use of protease inhibitors for accurate identification of specific proteolytic enzymes that are active in the stress response processes described in the present study.

## Figures and Tables

**Table 1:** *C. unicolor* DEGs Encoding for Putative Proteolytic and Proteasomal Activities Detected during Fungus Growth in Different Lighting Conditions.

Gene ID	SwissProt Best Hit	Expression		MEROPS ID
		Log2 Fold Change	*p*-Value	
**White Vs. Dark**				
XLOC_010853	Ubiquitin-like-specific protease 2 (1 × 10^−30^, sp|O13769|ULP2_SCHPO)	2.515418	3.71 × 10^−5^	C48.A09
XLOC_007208	E3 ubiquitin-protein ligase itt1 (4 × 10^−31^, sp|Q9US46|ITT1_SCHPO)	1.403588	0.017829	nd
XLOC_005837	Proteasomal ATPase-associated factor 1 (3 × 10^−28^, sp|Q9BRP4|PAAF1_HUMAN)	1.260725	0.013368	nd
XLOC_006576	Tripeptidyl-peptidase sed2 (1 × 10^−123^, sp|Q70J59|SED2_ASPFU)	1.129053	1.55 × 10^−5^	S53.010
XLOC_007257	Carboxypeptidase Y homolog A (6 × 10^−69^, sp|C0NX46|CBPYA_AJECG)	1.017356	0.003187	S10.001
XLOC_009122	Probable E3 ubiquitin-protein ligase hulA (0.0, sp|Q2UBP1|RSP5_ASPOR)	1.00622	0.019831	nd
XLOC_001799	Vacuolar membrane protease (3 × 10^−157^, sp|D8QAM0|PFF1_SCHCM)	0.868821	0.020832	nd
XLOC_001389	Probable ubiquitin carboxyl-terminal hydrolase 2 (1 × 10^−18^, sp|Q9P3U0|UBP2_SCHPO)	0.794248	0.046763	C19.A56
XLOC_007707	Ubiquitin carboxyl-terminal hydrolase 42 (5 × 10^−76^, sp|B2RQC2|UBP42_MOUSE)	0.590544	0.019906	nd
XLOC_002592	Extracellular metalloproteinase 2 (2 × 10^−104^, sp|Q6WIH2|MEP2_ARTBE)	0.586606	0.040586	nd
XLOC_011202	Probable ubiquitin-conjugating enzyme E2 W-A (9 × 10^−38^, sp|Q4VBH4|UB2WA_DANRE)	−0.57624	0.035189	nd
XLOC_003541	Ubiquitin-conjugating enzyme E2-20 kDa (6 × 10^−53^, sp|O00103|UBC11_SCHPO)	−0.63144	0.043634	nd
XLOC_006171	Ubiquitin-like protein ATG12 (6 × 10^−12^, sp|P0CM28|ATG12_CRYNJ)	−0.64219	0.040319	nd
XLOC_006914	Cytosol aminopeptidase (7 × 10^−70^, sp|Q68FS4|AMPL_RAT)	−0.65844	0.032525	nd
XLOC_007456	Ubiquitin-like protein SMT3 (4 × 10^−5^, sp|Q12306|SMT3_YEAST)	−0.65985	0.037187	nd
XLOC_009107	Serine protease inhibitor (8 × 10^−7^, sp|P81639|SPI_LENED)	−0.67441	0.042497	I66.001
XLOC_007328	Mitochondrial inner membrane protease subunit 1 (6 × 10^−18^, sp|O74800|IMP1_SCHPO)	−0.84674	0.03721	S26.002
XLOC_006577	Tripeptidyl-peptidase sed3 (1 × 10^−86^, sp|Q70GH4|SED3_ASPFU)	−0.89251	0.049589	S53.010
XLOC_001392	Mitochondrial intermediate peptidase (0.0, sp|Q6Y5M5|PMIP_PLEDJ)	−0.89328	0.021998	M03.006
XLOC_000740	Probable serine carboxypeptidase ARB_06414 (4 × 10^−32^, sp|D4AQA7|PEPS_ARTBC)	−0.98314	0.042819	S10.014
XLOC_007684	Ubiquitin carboxyl-terminal hydrolase 16 (1 × 10^−9^, sp|Q99LG0|UBP16_MOUSE)	−1.01978	0.032272	C19.050
XLOC_007692	Aspartic-type endopeptidase ctsD (0.0004, sp|Q4WNV0|CTSD_ASPFU)	−1.04223	0.030173	A01.077
XLOC_001024	Ubiquitin carboxyl-terminal hydrolase isozyme L3 (0.0004, sp|Q9JKB1|UCHL3_MOUSE)	−1.1451	0.002615	C12.003
XLOC_010540	Probable aspartic-type endopeptidase ARB_04018 (0.0002, sp|D4AIC4|Y4018_ARTBC)	−1.24835	0.02463	nd
XLOC_000692	Dipeptidyl aminopeptidase BIII (2 × 10^−6^, sp|V5YMB3|DAPB3_PSEMX)	−1.28209	0.032495	nd
XLOC_003799	Probable CAAX prenyl protease 2 (8 × 10^−11^, sp|O94448|RCE1_SCHPO)	−1.33829	0.011805	M79.002
XLOC_000739	Carboxypeptidase Y homolog ARB_02032 (5 × 10^−9^, sp|D4B0Q6|SCPD_ARTBC)	−1.4049	0.000481	S10.014
**Red Vs. Dark**				
XLOC_000446	Extracellular metalloproteinase MEP (4 × 10^−30^, sp|A4QWP8|MEP_MAGO7)	1.411536	0.001617	M36.001
XLOC_006786	Extracellular metalloprotease 1 (3 × 10^−32^, sp|C5P3X6|MEP1_COCP7)	1.36675	0.00231	M43.008
XLOC_006660	Serine protease inhibitor (6 × 10^−10^, sp|P81639|SPI_LENED)	1.063586	0.017117	I66.001
XLOC_006785	Extracellular metalloprotease UREG_07765 (7 × 10^−22^, sp|C4K014|MEP1_UNCRE)	0.92792	0.023814	nd
XLOC_005808	Proteasomal ATPase-associated factor 1 (3 × 10^−28^, sp|Q9BRP4|PAAF1_HUMAN)	0.921736	0.031266	nd
XLOC_006713	Signal peptidase complex catalytic subunit SEC11 (8 × 10^−91^, sp|B0D4L0|SEC11_LACBS)	0.677398	0.012642	S26.010
XLOC_010523	Carboxypeptidase Y homolog A (1 × 10^−75^, sp|C1GG77|CBPYA_PARBD)	0.606528	0.044429	S10.001
XLOC_007916	Peptidyl-Lys metalloendopeptidase (1 × 10^−75^, sp|Q9Y7F7|PLMP_ARMME)	-0.85908	0.049641	M35.004
**Blue Vs. Dark**				
XLOC_007257	Carboxypeptidase Y homolog A (6 × 10^−69^, sp|C0NX46|CBPYA_AJECG)	0.556057	0.017539	S10.001
XLOC_004719	Putative endoplasmic reticulum metallopeptidase 1 (1 × 10^−137^, sp|O94702|ERMP1_SCHPO)	0.526001	0.039009	nd
XLOC_004763	Putative endoplasmic reticulum metallopeptidase 1 (4 × 10^−17^, sp|O94702|ERMP1_SCHPO)	−0.6076	0.041892	nd
XLOC_000755	Ubiquitin-conjugating enzyme E2 15 (1 × 10^−65^, sp|Q9Y818|UBC15_SCHPO)	−0.6423	0.048532	nd
XLOC_004525	Probable serine carboxypeptidase ARB_06414 (9 × 10^−128^, sp|D4AQA7|PEPS_ARTBC)	−0.74374	0.042533	S10.014
XLOC_006786	Extracellular metalloprotease 1 (3 × 10^−32^, sp|C5P3X6|MEP1_COCP7)	−0.81067	0.011573	M43.008
XLOC_000384	Tripeptidyl-peptidase SED2 (9 × 10^−107^, sp|C5FBW2|SED2_ARTOC)	−0.86398	0.006162	nd
XLOC_007050	Ubiquitin (1 × 10^−36^, sp|P19848|UBIQ_COPCO)	−0.88446	0.03402	nd
XLOC_007970	Ubiquitin carboxyl-terminal hydrolase 18 (3 × 10^−7^, sp|Q67XW5|UBP18_ARATH)	−0.91702	0.033694	C19.A08
XLOC_011361	Ubiquitin-40S ribosomal protein S27a (8 × 10^−67^, sp|P14799|RS27A_NEUCR)	−1.02171	0.0153	nd
XLOC_007973	Extracellular metalloproteinase mep (1 × 10^−98^, sp|A1C4M2|MEP_ASPCL)	−1.06786	0.00057	M36.001
XLOC_007529	Aspartic protease (5 × 10^−73^, sp|O60020|ASPR1_PHARH)	−1.22239	0.005942	nd
**Green Vs. Dark**				
XLOC_005837	Proteasomal ATPase-associated factor 1 (3 × 10^−28^, sp|Q9BRP4|PAAF1_HUMAN)	0.91945	0.036747	nd
XLOC_010738	Cys-Gly metallodipeptidase dug1 (1 × 10^−136^, sp|Q9P6I2|DUG1_SCHPO)	0.760787	0.035739	M20.017
XLOC_011361	Ubiquitin-40S ribosomal protein S27a (8 × 10^−67^, sp|P14799|RS27A_NEUCR)	−0.92471	0.043594	nd
XLOC_007529	Aspartic protease (5 × 10^−73^, sp|O60020|ASPR1_PHARH)	−1.03688	0.028235	nd

nd—not determined in MEROPS database.

**Table 2:** Total proteolytic activities of acid and alkaline proteases measured against fluorogenic substrates (BODIPY-BSA, BODIPY-elastin and BODIPY-casein) detected in the culture extracts and clear supernatants of mycelium homogenates of *C. unicolor* growing in different lighting conditions.

Light	pH	Specific Activity [RFU/min/mg]				
		Incubation Time [day]				
		5	7	9	11	13
**Extracellular Acid Total Proteolytic Activity against BODIPY-BSA Substrate**						
dark	pH 3.5	37.86 ± 4.67	25.16 ± 1.57	23.01 ± 0.75	17.01 ± 2.10	17.82 ± 2.52
white	pH 3.5	33.06 ± 3.56	29.57 ± 2.33	24.92 ± 1.40	18.52 ± 2.55	19.27 ± 2.71
red	pH 3.5	41.05 ± 3.29	35.17 ± 2.99	27.74 ± 2.98	18.68 ± 2.98	24.06 ± 2.84
green	pH 3.5	28.08 ± 1.52	28.14 ± 2.01	26.66 ± 2.89	19.78 ± 2.39	21.68 ± 2.49
blue	pH 3.5	37.72 ± 2.80	31.03 ± 3.44	28.07 ± 2.84	22.07 ± 1.99	19.87 ± 1.99
**Intracellular Acid Total Proteolytic Activity against BODIPY-BSA Substrate**						
dark	pH 3.5	24.75 ± 1.24	19.80 ± 0.99	14.54 ± 0.73	12.80 ± 0.64	15.96 ± 0.80
white	pH 3.5	24.95 ± 1.25	14.73 ± 0.74	17.91 ± 0.90	14.14 ± 0.74	14.19 ± 0.71
red	pH 3.5	31.02 ± 1.55	21.47 ± 1.07	21.15 ± 1.06	16.36 ± 0.82	16.21 ± 0.81
green	pH 3.5	16.35 ± 0.82	23.48 ± 1.17	15.88 ± 0.79	14.14 ± 0.71	17.58 ± 0.88
blue	pH 3.5	22.76 ± 1.14	21.74 ± 1.09	13.97 ± 0.70	11.96 ± 0.60	16.73 ± 0.84
**Extracellular Alkaline Total Proteolytic Activity against BODIPY-BSA Substrate**						
dark	pH 8.0	39.04 ± 4.79	26.63 ± 0.71	23.65 ± 1.32	14.91 ± 1.74	16.26 ± 2.28
white	pH 8.0	43.48 ± 6.34	31.59 ± 1.94	24.21 ± 2.16	16.88 ± 2.50	15.15 ± 2.66
red	pH 8.0	52.71 ± 7.18	34.38 ± 2.52	22.21 ± 1.00	12.33 ± 0.72	14.35 ± 1.33
green	pH 8.0	39.11 ± 6.08	36.61 ± 2.67	26.73 ± 2.78	20.27 ± 1.76	17.69 ± 2.81
blue	pH 8.0	51.34 ± 6.62	26.85 ± 5.24	18.01 ± 1.59	12.30 ± 2.04	11.28 ± 1.50
**Intracellular Alkaline Total Proteolytic Activity against BODIPY-BSA Substrate**						
dark	pH 8.0	31.27 ± 1.56	31.69 ± 1.58	29.86 ± 1.49	23.29 ± 1.16	24.08 ± 1.20
white	pH 8.0	24.73 ± 1.24	19.18 ± 0.96	21.48 ± 1.07	17.06 ± 0.85	21.28 ± 1.06
red	pH 8.0	37.99 ± 1.90	34.40 ± 1.72	31.76 ± 1.59	30.97 ± 1.55	26.87 ± 1.34
green	pH 8.0	29.54 ± 1.48	34.42 ± 1.72	31.29 ± 1.56	29.77 ± 1.49	28.98 ± 1.45
blue	pH 8.0	29.82 ± 1.49	32.48 ± 1.62	31.06 ± 1.55	23.06 ± 1.15	26.38 ± 1.32
**Extracellular Elastase-like Proteolytic Activity**						
dark	pH 8.0	22.34 ± 1.57	18.60 ± 3.77	8.30 ±2.36	7.52 ± 1.59	10.98 ± 0.99
white	pH 8.0	21.18 ± 8.80	24.68 ± 10.17	29.26 ± 5.26	16.57 ± 6.33	14.98 ± 7.49
red	pH 8.0	72.61 ± 28.79	64.22 ± 21.55	50.62 ± 19.87	44.54 ± 16.17	41.75 ± 18.31
green	pH 8.0	32.52 ± 5.22	18.40 ± 0.02	20.65 ± 0.12	8.52 ± 0.85	15.82 ± 2.51
blue	pH 8.0	68.93 ± 26.53	61.73 ± 21.27	60.29 ± 25.20	41.35 ± 17.40	28.95 ± 18.40
**Intracellular Elastase-like Proteolytic Activity**						
dark	pH 8.0	13.09 ± 0.65	8.26 ± 0.41	2.91 ± 0.15	2.13 ± 0.11	2.15 ± 0.11
white	pH 8.0	15.97 ± 0.80	15.48 ± 0.77	11.46 ± 0.57	11.01 ± 0.55	13.29 ± 0.66
red	pH 8.0	58.74 ± 2.94	52.10 ± 2.61	51.23 ± 2.56	45.52 ± 2.28	46.60 ± 2.33
green	pH 8.0	12.61 ± 0.63	12.77 ± 0.64	9.78 ±0.49	4.47 ± 0.22	6.61 ± 0.33
blue	pH 8.0	57.69 ± 2.88	57.47 ± 2.87	53.15 ± 2.66	49.18 ± 2.46	59.79 ± 2.99
**Extracellular Caseinolytic Activity**						
dark	pH 8.0	156.62 ± 0.66	127.34 ± 3.01	112.62 ± 2.23	89.35 ± 1.17	95.53 ± 3.40
white	pH 8.0	165.83 ± 0.57	128.20 ± 3.81	126.95 ± 2.59	98.59 ± 1.96	98.23 ± 4.53
red	pH 8.0	19.87 ± 3.63	24.37 ± 2.86	15.97 ± 0.22	7.71 ± 0.08	12.17 ± 6.83
green	pH 8.0	153.64 ± 2.89	136.88 ± 2.46	145.94 ± 0.21	95.40 ± 1.46	111.86 ± 1.19
blue	pH 8.0	29.74 ± 0.93	16.52 ± 4.66	22.99 ± 5.00	7.43 ± 3.71	9.10 ± 4.55
